# Acute Cholelithiasis With Acute Pancreatic Calcifications: A Unique Presentation of Sickle Cell Crisis

**DOI:** 10.7759/cureus.30272

**Published:** 2022-10-13

**Authors:** Nidhi Popat, Sunil Kumar, Bhavik S Unadkat

**Affiliations:** 1 Department of Medicine, Jawaharlal Nehru Medical College, Datta Meghe Institute of Medical Sciences (DU), Wardha, IND; 2 Department of Radiology, Jawaharlal Nehru Medical College, Datta Meghe Institute of Medical Sciences (DU), Wardha, IND

**Keywords:** cholangiopathy, vaso-occlusive crisis, sickle cell crisis, acute pancreatic calcifications, calcific pancreatitis, acute cholelithiasis

## Abstract

Sickle cell disease is one of the most prevalent hemoglobinopathies around the world. Sickle cell anemia manifests due to a genetic alteration caused by substituting a single nucleotide in place of another nucleotide in the beta-globin gene. This mutation causes rigidity and sickling of the erythrocytes when the oxygen saturation falls in conditions like infection, dehydration, stress, etc. The distorted erythrocytes cause vaso-occlusion, tissue ischemia, and infarction. Cholelithiasis is known to be a frequent complication in cases of sickle cell anemia. It occurs because of chronic hemolysis. In sickle cell patients, calcific pancreatitis is an infrequent complication of vaso-occlusive crisis. It appears in connection with cholelithiasis in most patients. This case report highlights a rare presentation of sickle cell crisis complicated with multiple pigmented gall bladder calculi and pancreatic calcifications.

## Introduction

Sickle cell disease is an autosomal recessive hemoglobinopathy, which occurs due to a point mutation in the beta-globin chain of hemoglobin, leading to the production of hemoglobin S. The adult hemoglobin is a heterotetramer comprising four hemoglobin chains: two α chains and two β chains (HbAA). Sickle cell hemoglobinopathy occurs because of a single modification of the valine amino acid in the place of glutamate at the sixth place in the β hemoglobin gene. The inheritance of two sickle β hemoglobin genes (HbSS) leads to sickle cell anemia [1].

The tropics, particularly Sub-Saharan Africa, the Middle East, and India, are where sickle cell disease is most prevalent. The frequency of sickle cell disease in India has extended from 9.4% to 22.2% over time in endemic areas. India alone accounts for almost 50% of all sickle cell cases worldwide [2].

Sickle cell disease has the potential to affect multiple organ systems in the body. Essentially, every abdominal organ can get involved in a sickle cell crisis due to sickling, stasis, hypercoagulability, and capillary engorgement in the larger vessels [3]. Each year, 10% of sickle cell patients get hospitalized because of severe abdominal pain [4]. The most common causes of this acute abdominal pain are acute cholecystitis, cholelithiasis, ischemic bowel, hepatic sequestration, splenic sequestration, renal papillary necrosis, peptic ulcer disease, urinary tract infection, etc. [5]. The hepatobiliary system is most commonly involved in sickle cell disease. Cholelithiasis is known to be a common complication in sickle cell crises. It occurs because of chronic hemolysis. Calcific pancreatitis is an infrequent complication of vaso-occlusive sickle cell crisis in sickle cell patients. It occurs in connection with cholelithiasis in most patients [4]. The symptoms are identical to other etiologies of acute abdominal pain. Therefore, it becomes a diagnostic dilemma. The diagnosis is made on the grounds of clinical suspicion or biochemical evidence, but the chief modality is radiographic studies.

Here, we report a rare presentation of multiple gall bladder calculi and pancreatic calcifications in an 18-year-old male, a known case of sickle cell disease.

## Case presentation

An 18-year-old male patient with a known case of homozygous sickle cell anemia presented to casualty with the chief complain of severe abdominal pain for the last four days. The pain as described by the patient was diffuse but more severe in the upper-right quadrant, intermittent type, dull aching, acute in onset, progressive in nature, radiating to back, aggravated on movement, and not relieved on taking oral medications for pain. It was associated with yellowish discoloration of skin and sclera. The patient underwent a blood transfusion with one unit of packed red cells two days prior, because of anemia. There was no history of any other comorbidities.

**Figure 1:**
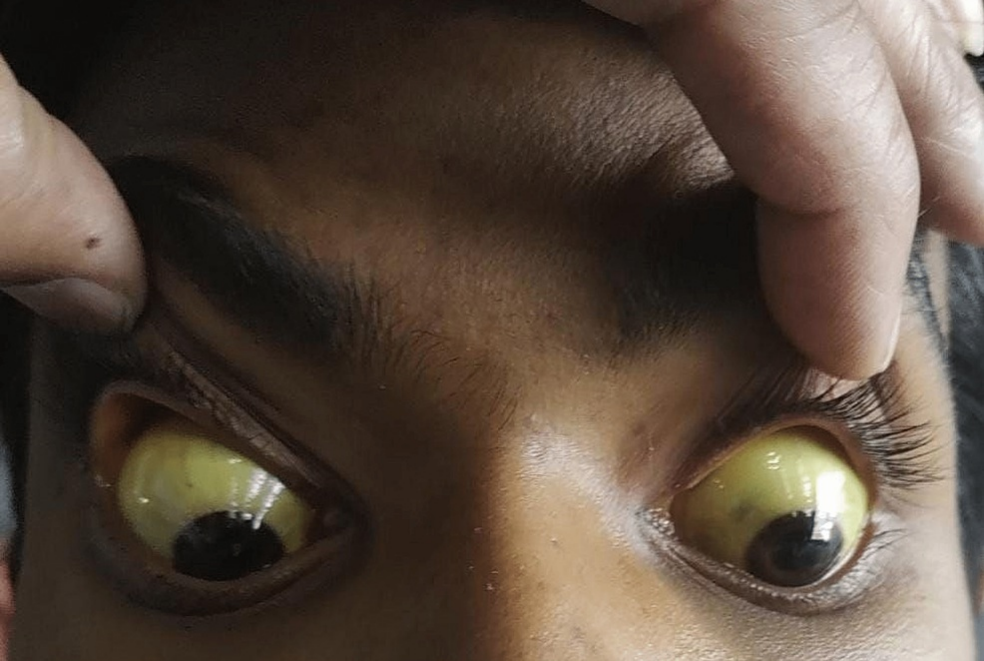
Scleral icterus

On examination, the patient was well oriented to time, place, and person. He was afebrile, pulse rate of 88 bpm, regular rhythm, and normal volume. The patient’s respiratory rate was 18 per minute, his blood pressure was 130/80 mmHg, his oxygen saturation was 99% on room air, and scleral icterus was present (Figure 1). Chest sounds were clear on both sides, heart sounds were normal, and there was no focal neurological deficit. Per abdominal examination elicited tenderness in the right upper quadrant and hepatosplenomegaly. No evidence of rigidity, guarding, or rebound tenderness. Bowel sounds were heard normally.

Investigations

All baseline lab investigations were advised (Table 1).

**Table 1:** Laboratory investigations Lab investigations showed raised amylase and lipase levels and a deranged liver function test.

Laboratory Investigations	Measured values
Complete Blood Count	Hemoglobin: 8.2 gm/dl
	Mean corpuscular volume: 76.9 fl
	Total leukocyte count: 8,200/μL
	Platelet count: 1.06 lakhs/mm^3^ (lab reference range: 1.5-4.5 lakhs/mm^3^, (Reticulocyte count increased by 7.1%))
Peripheral smear	Red blood cells (RBC): normocytic normochromic with mild anisopoikilocytosis showing few microcytic hypochromic RBCs and pencil cells.
	Platelets: reduced on smear
Sickling test (early and late)	Early: negative
	Late: positive
Liver function test	Alkaline Phosphatase: 211 IU/L (normal levels: 35-130)
	Alanine aminostransferase: 117 IU/L (normal levels: 5-40)
	Aspartate aminotransferase: 143 IU/L (normal levels: 5-40)
	Total bilirubin: 11.7 (normal levels: 0.1-1.2 mg/dl)
Amylase	1672 U/L (normal levels: 40-140 U/L)
Lipase	7954 U/L (normal levels: 0-160 U/L)
Lactate dehydrogenase (LDH)	179 U/L (normal levels: 140-280 U/L)
Renal function test	Urea: 28 mg/dl (normal range: 7-25 mg/dl)
	Serum creatinine: 0.7mg/dl (normal levels: 0.6 -1.1 mg/dl)
	Sodium: 141 mEq/L (normal levels: 135-145 mEq/L)
	Potassium: 3.1 mmol/L (normal levels: 3.5-5.1 mmol/L)

An ultrasonography scan of the abdomen and pelvis was performed. It was suggestive of the following findings (Table 2).

**Table 2:** Ultrasonography abdomen pelvis findings of the case

Organ	Findings
Gall bladder	The gall bladder shows multiple calculi, the largest measuring 16mm; the gall bladder wall is thick and edematous. (Figure 2)
Pancreas	The pancreas appears heterogeneous and bulky, with parenchymal calcifications suggestive of pancreatitis. –few foci of calcification were seen in the main pancreatic duct as well. (Figure 3)

**Figure 2:**
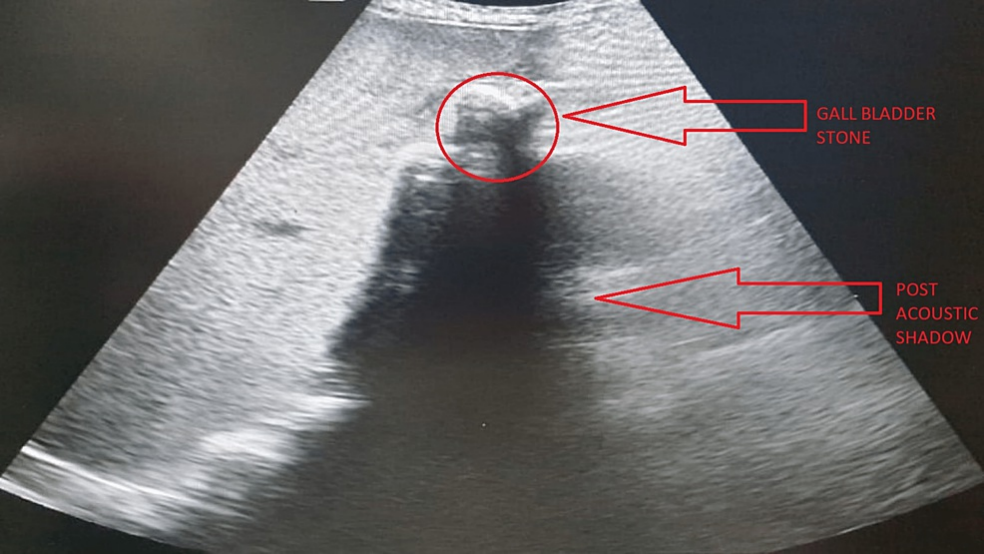
Ultrasonography image showing calculus in the gall bladder

**Figure 3:**
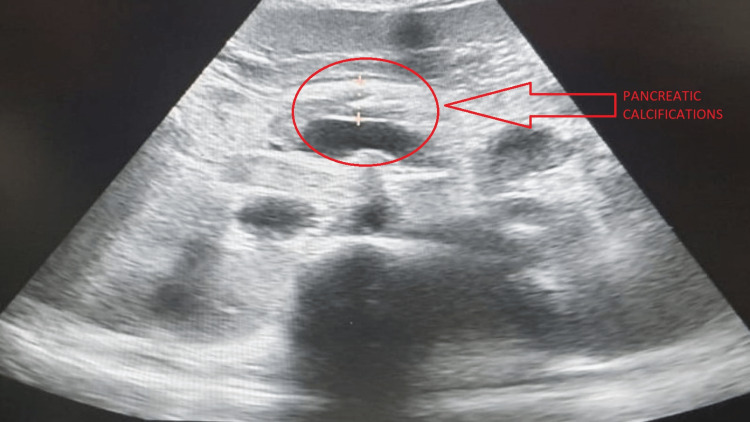
Ultrasonography image showing pancreatic calcifications

Ultrasonography of the abdomen and pelvis was suggestive of hepatomegaly, splenomegaly, cholelithiasis (Figure 2), bulky heterogenous pancreas with parenchymal calcifications (Figure 3) suggestive of pancreatitis, and gross ascites.

Treatment

After admission, immediate intravenous access, and fluid resuscitation were initiated, and the patient was put on broad-spectrum antibiotics (injection of ceftriaxone 1 gm intravenously twice daily, tablet rifaximin 550 mg twice daily, and tablet nitrofurantoin 200 mg twice daily). Injection of tramadol 100 mg in 100 ml of normal saline was given for pain management. Tablet hydroxyurea 500 mg and tablet ursodeoxycholic acid 300 mg were prescribed twice daily. Tablet of folic acid 5 mg was given once daily. The patient underwent an endoscopic retrograde cholangiopancreatography (ERCP), which revealed calcifications in the pancreas and biliary tree without any evidence of obstruction. The patient was treated with an ERCP-guided pancreatic duct stenting and common bile duct stenting. The patient was started on antibiotics post-operatively and was transfused several units of hemoglobin S-free blood. The pancreatic and liver enzymes started to normalize within a week of surgery.

Outcome

After the surgery, the patient’s bilirubin, amylase, and lipase levels were reduced, and the rest of the course in the hospital was uneventful. The patient’s condition improved gradually, and he was discharged after 27 days of admission. The patient is currently stable and doing well on follow-up.

## Discussion

Chicago cardiologist James B. Herrick initially recognized sickle cells in the year 1904 [4]. In 1922, Vernon Mason coined the term "sickle-cell anemia" [4]. Linus Pauling, in the year 1949, was the first to exhibit that a genetic defect in the hemoglobin molecule leads to sickle cell anemia [4]. Currently, it is established that sickle cell anemia occurs due to the point mutation of the amino acid valine in place of glutamate in the sixth position in the beta hemoglobin chain consisting of 146 amino acids [4]. This causes hemoglobin S production. This hemoglobinopathy gets transmitted as a mendelian dominant gene. The inheritance of HbSS leads to the manifestation of clinically overt sickle cell anemia.

This point mutation in sickle cell disease causes polymerization of the hemoglobin molecule when the oxygen levels in the body fall. This leads to distortion of the shape of the red blood cells (in-vivo sickling) and microvascular occlusion. These processes, along with their subsequent effects like an inflammatory response, reperfusion injury, and cellular dehydration, which are crucial pathophysiological mechanisms, lead to the various systemic complications of sickle cell crisis. In patients with HbSS patterns, there are four different types of crises that bring them to medical attention: vascular occlusion crisis, hemolytic crisis, sequestration crisis, and aplastic crisis [3].

The hepatobiliary system is one of the primary systems affected by sickle cell disease. Some processes, such as sinusoidal blockage by sickled red cells (causing ischemia of hepatocytes and subsequent ballooning of neighboring liver cells), and intra-canalicular cholestasis, may help to explain the pathophysiology of the hepatobiliary complications in sickle cell disease. Acute hepatic sequestration occurs due to vaso-occlusion caused by the trapping of platelets and red blood cells in the liver. Based on the degree of severity of hepatocyte ischemia, cholestasis, and trapping of RBCs and platelet, the sickle cell crisis can manifest in the form of acute hepatic crisis, acute hepatic sequestration, or sickle cell intrahepatic cholestasis. Moreover, cholangiopathy occurs due to bile duct ischemia secondary to sickling. Cholelithiasis occurs either directly due to the sickling process or indirectly due to chronic hemolysis, which leads to unconjugated hyperbilirubinemia [4,6].

Such patients report to the hospital with variable severity of abdominal pain. The pain is usually diffuse, acute in onset, sharp or colicky, and duration may last a few to 48 hours. This classical pain of sickle cell crisis occurs due to vaso-occlusive episodes, which may mimic an acute abdomen condition. Jaundice, abdominal pain, elevated liver enzymes, and leucocytosis usually occur in 10% of all patients having an HbSS pattern (two sickle β hemoglobin genes). If present simultaneously, all these symptoms are referred to as liver or hepatic crises. There are multiple hepatobiliary complications of sickle cell disease, such as cholelithiasis, choledocholithiasis, pancreatitis, viral hepatitis, cirrhosis, intrahepatic cholestasis, hepatic hemosiderosis, pseudocyst of the pancreas, portal hypertension, fulminant hepatitis, etc. [3,7-9]. Patients with such liver crises can present with clinical and laboratory findings which are very similar and very difficult to differentiate, often creating a diagnostic dilemma for the physician [3].

In sickle cell patients, cholelithiasis is a known complication. The prevalence of cholelithiasis is as high as 67% in sickle cell crises [3]. This prevalence increases progressively with age. These stones are often calcium bilirubinate pigment stones. Patients with sickle cell disease form gallstones more frequently and at a younger age than others. This is because of chronic hemolysis, which causes unconjugated hyperbilirubinemia due to heme catabolism, bilirubin precipitation, and the development of bilirubinate crystals or irregularities in the functioning of the gall bladder or the bile acid metabolism. 

The liver excretes this excessive bilirubin in the bile. This bile from the gall bladder is a highly saturated solution composed chiefly of cholesterol and hemoglobin derivatives. If there is an elevation in the levels of these cholelithogenic components, it can cause precipitation leading to bilirubinate cholelithiasis. Other causes for the formation of these gallstones could be infection, stasis, etc. In patients of sickle cell crisis presenting with cholelithiasis, raised levels of indirect bilirubin, reticulocytes, and hemoglobin-F suggests that the hemolysis rate is the primary determinant factor for gallstone formation [5]. Cholelithiasis may further lead to cholecystitis, cholangitis, choledocholithiasis, and gallstone pancreatitis, which can lead to excessive morbidity in sickle cell crises. In these patients, around 50% of these gallstones appear on plain films. This is because these black stones are composed of calcium bilirubinate, which makes them radio-opaque [5]. In sickle cell patients presenting with cholelithiasis, the treatment of choice is cholecystectomy. Laparoscopic cholecystectomy is preferred, being the minimally invasive technique. It is cosmetically better, the patients are advised a shorter hospital stay, and the postoperative pain is also minimum [4,10].

Along with severe abdominal pain, our patient had significantly raised levels of serum amylase and lipase. In addition to that, he also had ultrasonography findings suggestive of pancreatic calcification.

## Conclusions

In sickle cell patients, calcific pancreatitis is an infrequent complication of vaso-occlusive crisis. It can take place in association with cholelithiasis. Occlusion of small vessels and ischemia to the pancreas could be the cause in such cases. There was no evidence of drug, toxin, trauma, any obstructive etiology, or alcohol intake in our patient, thereby indicating that the calcific pancreatitis is most probably due to an ischemic etiology as a result of sickling. This case report mainly focuses on appealing the clinicians to evaluate the patients of sickle cell anemia for pancreatic complications to avoid further morbidity and mortality.
